# Molecular Epidemiology of Hypervirulent Carbapenemase-Producing *Klebsiella pneumoniae*

**DOI:** 10.3389/fcimb.2021.661218

**Published:** 2021-04-07

**Authors:** Dakang Hu, Yuming Li, Ping Ren, Dongxing Tian, Wenjie Chen, Pan Fu, Weiwen Wang, Xiaobin Li, Xiaofei Jiang

**Affiliations:** ^1^ Department of Laboratory Medicine, Huashan Hospital, Fudan University, Shanghai, China; ^2^ Department of Intensive Care Unit, Huashan Hospital, Fudan University, Shanghai, China; ^3^ Zhejiang Provincial Demonstration Center of Laboratory Medicine Experimental Teaching, Wenzhou Medical University, Wenzhou, China; ^4^ Department of Infectious Diseases, Huashan Hospital, Fudan University, Shanghai, China; ^5^ Department of Microbiology, Children’s Hospital of Fudan University, Shanghai, China; ^6^ Zhuhai Precision Medical Center, Zhuhai People’s Hospital (Zhuhai Hospital Affiliated With Jinan University), Zhuhai, China

**Keywords:** *Klebsiella pneumoniae*, virulence, plasmid, *bla*_KPC_, epidemiology

## Abstract

**Objective:**

To investigate the overall distributions of key virulence genes in *Klebsiella pneumoniae*, especially the hypervirulent *bla*_KPC_-positive *K. pneumoniae* (Hv-*bla*_KPC_(+)-KP).

**Methods:**

A total of 521 complete genomes of *K. pneumoniae* from GenBank were collected and analyzed. Multilocus sequence typing, molecular serotyping, antibiotic-resistance, virulence genes and plasmid replicon typing were investigated.

**Results:**

Positive rates of virulence genes highly varied, ranging from 2.9 (*c-rmpA/A2*) to 99.6% (*entB*). Totally 207 strains presented positive *fimH*, *mrkD*, *entB* and *wzi* and 190 showed positive *fimH*, *mrkD*, *entB*, *irp2* and *wzi*, which were the two primary modes. A total of 94, 165 and 29 strains were denoted as hypervirulent *K. pneumoniae* (HvKP), *bla*_KPC_(+)-KP and Hv-*bla*_KPC_(+)-KP. ST11 accounted for 17 among the 29 Hv-*bla*_KPC_(+)-KP strains; Genes *iucA*, *p-rmpA2* and *p-rmpA* were positive in 28, 26 and 18 Hv-*bla*_KPC_(+)-KP strains respectively. Among the 29 Hv-*bla*_KPC_(+)-KP strains exhibiting four super clusters from GenBank, IncHI1B plasmids carrying virulence genes and IncFII ones with *bla*_KPC_ were responsible for both 23 strains respectively.

**Conclusions:**

Positive rates of virulence genes vary remarkably in *K. pneumoniae*. Genes *iucA*, *p-rmpA2* and *p-rmpA* were primary ones inducing Hv-*bla*_KPC_(+)-KP. IncHI1B plasmids carrying virulence genes and IncFII ones with *bla*_KPC_ constitute the primary combination responsible for Hv-*bla*_KPC_(+)-KP. The making of Hv-*bla*_KPC_(+)-KP is mostly *via bla*_KPC_(+)-KP acquiring another plasmid harboring virulence genes.

## Introduction

*Klebsiella pneumoniae*, a ubiquitous and an opportunistic pathogen, can induce both nosocomial and community-acquired infections (Russo and Marr, 2019; Choby et al., 2020). The former consist of pneumonia, bacteremia, urinary tract infections, etc. The latter include pyogenic liver abscess, endophthalmitis, meningitis, necrotizing fasciitis, etc. *K. pneumoniae* inducing such “invasive syndrome” is termed as hypervirulent *K. pneumoniae* (HvKP), which is more virulent than “classical” *K. pneumoniae* (cKP) typically responsible for nosocomial infections (Russo and Marr, 2019). Many virulence factors are involved in such pathogenesis, e.g. capsule, lipopolysaccharide, Types 1 and 3 fimbriae, siderophores, allantoin metabolism, etc. (Paczosa and Mecsas, 2016). Further, numerous genes are determinants of those factors. Genes *p-rmpA*, *p-rmpA2* and *c-rmpA/A2* all could induce hypercapsule (Paczosa and Mecsas, 2016). Traditionally, HvKP was usually susceptible to most antibiotics except inherently resistant ampicillin (Fang et al., 2007).

With years passing, *K. pneumoniae*, regardless of cKP or HvKP, becomes more and more drug-resistant, among which carbapenem-resistance is of great concern. Carbapenem-resistance is mostly conferred by carbapenemase gene (*bla*_KPC_), New Delhi metallo-β-lactamase gene (*bla*_NDM_), and oxacillinases-48 gene (*bla*_OXA-48_), which are predominantly carried on the mobile genetic elements (Zhang et al., 2015; Lee et al., 2016). Among them, *bla*_KPC_, particularly *bla*_KPC-2/3_ is predominant (Kopotsa et al., 2019). Carbapenem-resistant *K. pneumoniae* (CRKP) has now become a great public health threat worldwide (Lee et al., 2016; Niu and Li, 2019), due to its causing high mortality and medical burden.

In the past decades, hypervirulence and drug-resistance advance separately in *K. pneumoniae*. CRKP was not usually considered hypervirulent (Zhang et al., 2017a). However, their convergence was found in recent years worldwide (Zhang et al., 2015; Lam et al., 2019; Wozniak et al., 2019). Not surprisingly, such *K. pneumoniae* strains could induce an overwhelming mortality (Gu et al., 2018). Due to the mobility of elements carrying virulence and drug-resistance genes, hypervirulent carbapenem-resistant *K. pneumoniae* (Hv-CRKP) gained more and more prevalence with its positive rate reaching 7.4–15.0% among CRKP in recent years (Lee et al., 2017). To date, the overall distribution of key virulence genes in *K. pneumoniae* strains, in particular hypervirulent *bla*_KPC_-positive *K. pneumoniae* (Hv-*bla*_KPC_(+)-KP), was rarely reported. Here, we collected 521 K*. pneumoniae* strains from GenBank. Upon the yielded data, we could get insight into the distributions of key virulence genes in *K. pneumoniae*, particularly Hv-*bla*_KPC_(+)-KP.

## Materials and Methods

### *K. pneumoniae* Strains

A total of 521 complete whole genomes (**Table S1**) of *K. pneumoniae* from the GenBank Database (https://www.ncbi.nlm.nih.gov/genome/815; download date: May 13th, 2020) were analyzed in this study. Those draft genomes (contigs and scaffolds) were not included. The 521 strains included 28.4% (148 strains) from Mainland China, 4.4% (23 strains) from Taiwan of China, 1.5% (eight strains) from Hong Kong of China, 25.7% (134 strains) from USA, 9.6% (50 strains) from Australia, 6.7% (35 strains) from UK, 3.8% (20 strains) from Germany, 2.7% (14 strains) from Korea, 2.3% (12 strains) from India, 2.1% (11 strains) from France, 1.5% (eight strains) from Japan and 11.1% (58 strains) from other countries.

### Multilocus Sequence Typing (MLST)

The DNA fasta sequences of the 521 genomes were compared with the *K. pneumoniae* MLST database (Larsen et al., 2012) containing the seven housekeeping genes (*gapA*, *infB*, *mdh*, *pgi*, *phoE*, *rpoB* and *tonB*) and the STs were yielded.

### Determination of Serotypes, Antibiotic-Resistance and Virulence Genes

For the genomes of *K. pneumoniae* from GenBank, the accession numbers were directly used to determine the capsular types *via* the database of Institute Pasteur (https://bigsdb.pasteur.fr/klebsiella/klebsiella.html). The potential *beta*-lactamase genes were determined using the Resfinder software version 3.2 (https://cge.cbs.dtu.dk/services/ResFinder/) (Zankari et al., 2012) with the minimum coverage of 60% and minimum identity of 90%, and the virulence genes were predicted using NCBI_BLAST (megablast) searches against the virulence genes of *K. pneumoniae* with experimental supports (**Table S2**) with the cut-off coverage of 80% and cut-off identity of 80%.

For virulence genes in this study, they could be classified as the following categories: metabolism (*peg-344*), colonization (*allS*), assembling channel protein for capsular polysaccharides or macromolecular exopolysaccharides (EPS, *wzy-K1*), regulator of mucoid phenotype (*p-rmpA2*, *c-rmpA/A2*, *p-rmpA*), Type 1 fimbriae (*fimH*), Type 3 fimbriae (*mrkD*), enterobactin (*entB*), yersiniabactin (*irp2*), salmochelin (*iroN*), and aerobactin (*iucA*) and capsular polysaccharide-anchor (*wzi*).

### Determination of HvKP, cKP and Hv-*bla*_KPC_(+)-KP

The factors responsible for HvKP include hypercapsule (by *p-rmpA2*, *c-rmpA/A2*, *p-rmpA*), EPS (by *wzy-K1*) and excessive siderophores (Paczosa and Mecsas, 2016; Russo and Marr, 2019). In this study, HvKP could be defined as: positive *wzy-K1*, ≥3 positive siderophore genes (*entB*, *irp2*, *iroN* and *iucA*), or ≥1 positive capsule-regulating genes (*p-rmpA2*, *c-rmpA/A2* and *p-rmpA*). Non-HvKP is termed as cKP. Hv-*bla*_KPC_(+)-KP is defined as HvKP carrying *bla*_KPC_.

### Phylogenetic Analysis and Plasmid Replicon Analysis

The phylogenetic tree of *K. pneumoniae* strains was generated using kSNP3 (Gardner et al., 2015) software for *K. pneumoniae* chromosomes and displayed by iTOL (Letunic and Bork, 2016) with midpoint rooting. For the plasmids, the phylogenetic patterns were based on the presence/absence of orthologous gene families of all the plasmids under analysis. A binary gene presence/absence matrix was created using OrthoFinder (Emms and Kelly, 2019) with default settings and a hierarchical cluster result was shown by iTOL (Letunic and Bork, 2016).

Plasmid replicon typing was determined using the PlasmidFinder software version 2.0.1 with the minimum coverage of 60% and minimum identity of 95%.

### Statistical Analysis

Statistical analysis was performed using GraphPad Prism 8 software (GraphPad Software Inc., USA). Chi-square test was used to analyze comparisons between groups; *p <*0.05 was considered statistically significant.

## Results

### Distributions of Virulence Genes and Predicted Key Virulence Factors

**Figure 1:**
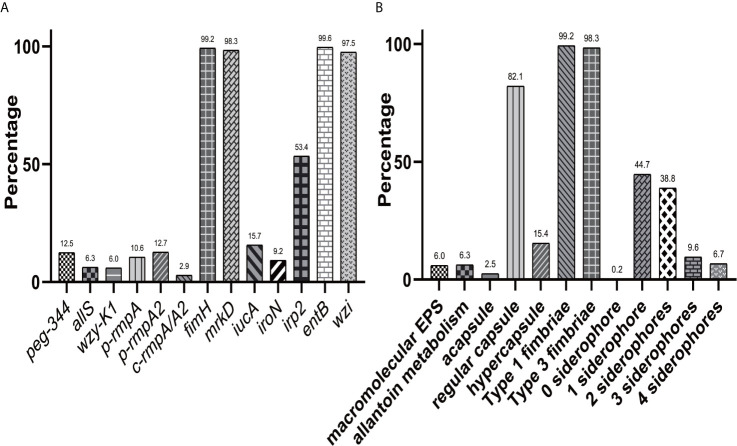
Distributions of virulence genes and factors in *K. pneumoniae*. **(A)** Distribution of 13 virulence genes in 521 K*. pneumoniae* strains. **(B)** Distribution of virulence factors in 521 *K. pneumoniae* strains.

**Figure 2:**
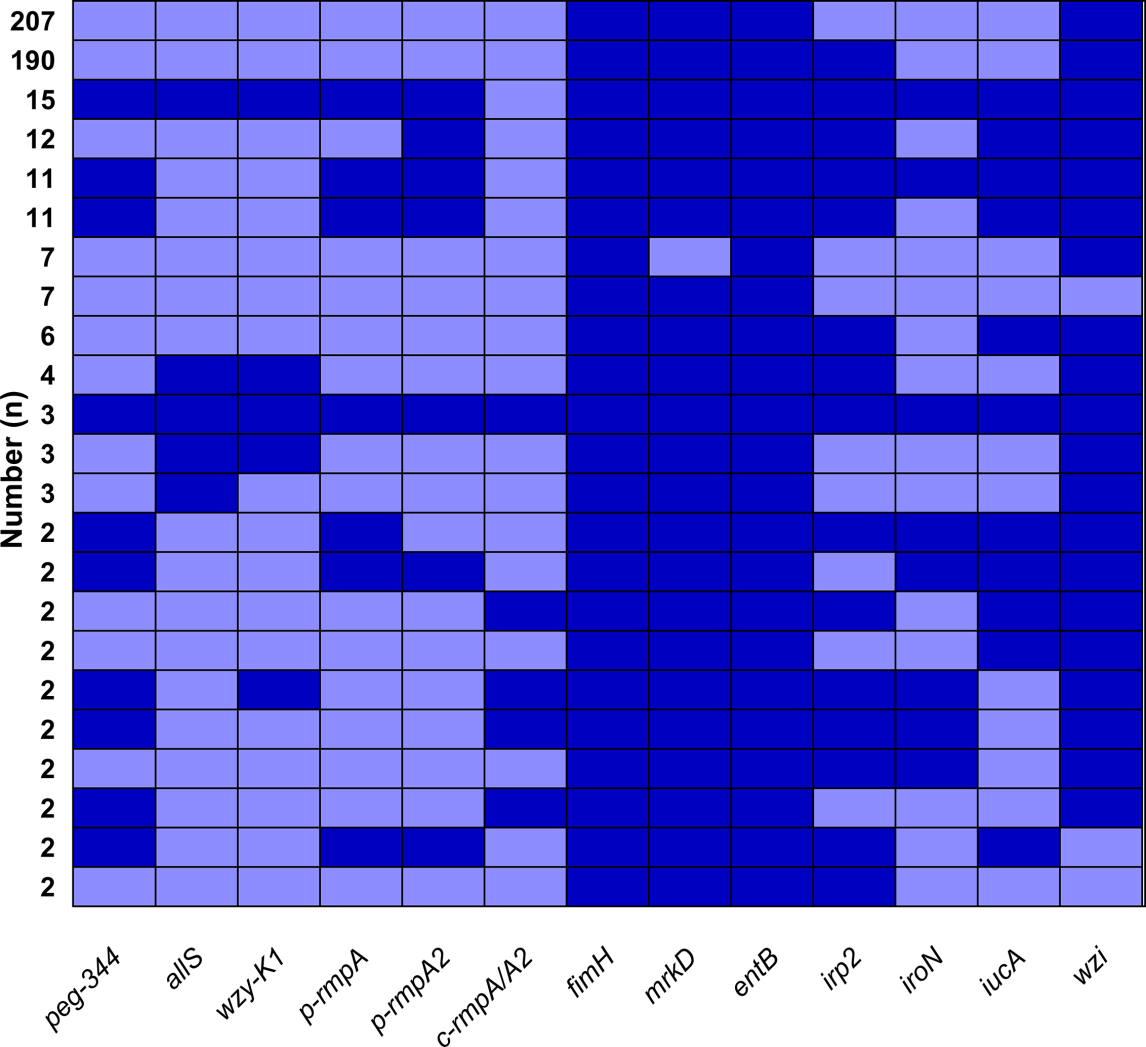
Modes of virulence genes in 521 *K. pneumoniae* strains. The presence of virulence genes is represented by a dark blue box and the absence of others is represented by a light blue box. Only those with ≥ 2 strains were included in **Figure 2**.

**Figure 1A** showed overwhelmingly different positive rates of virulence genes, ranging from 2.9 (*c-rmpA/A2*) to 99.6% (*entB*) among the 521 *K. pneumoniae* strains. Four genes (*fimH*, *mrkD*, *entB* and *wzi*) exhibited prevalence rates of > 90.0%, 1 (*irp2*) > 50.0% and the others < 25.0%. For the *rmpA*s, the order was: *p-rmpA2* (12.5%), *p-rmpA* (10.6%) and *c-rmpA/A2* (2.9%). For the four siderophore genes, the order was: *entB* (99.6%), *irp2* (53.4%), *iucA* (15.7%) and *iroN* (9.2%). Positive rates of *iroN* and *iucA* were both lower than that of *irp2* and *entB* (all *p <* 0.0001). **Figure 1B** presented different positive rates of predicted virulence factors, ranging from 0.2% (none siderophore) to 99.2% (Type 1 fimbriae). The factors (Types 1 and 3 fimbriae, regular capsule, one or two siderophores) were found more common; 436 (83.7%) strains were found possessing ≤ 2 siderophores. **Figure 2** showed 23 modes of virulence genes in *K. pneumoniae*: each ≥2 strains. Totally 207 strains presented positive *fimH*, *mrkD*, *entB* and *wzi* and 190 showed positive *fimH*, *mrkD*, *entB*, *irp2*, and *wzi* simultaneously, which were the two primary modes and accounted for 39.7% and 36.5% respectively.

**Figure 3:**
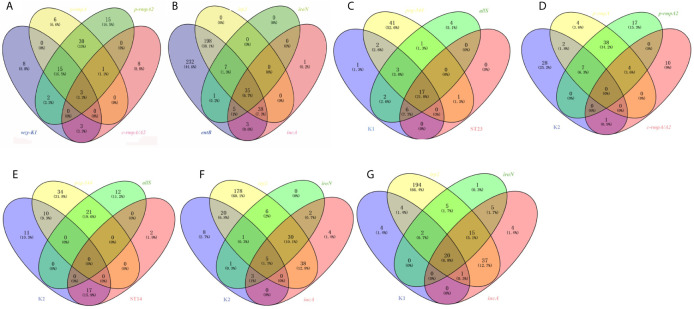
Venn diagrams of various relationships among virulence genes, serotypes and ST types. **(A)** Venn diagram of *wzy-K1*, *p-rmpA*, *p-rmpA2* and *c-rmpA/A2*. **(B)** Venn diagram of *entB*, *irp2*, *iroN* and *icuA.* **(C)** Venn diagram of K1, *peg-344*, *allS* and ST23. **(D)** Venn diagram of K2, *p-rmpA*, *p-rmpA2* and *c-rmpA/A2.* **(E)** Venn diagram of K2, *peg-344*, *allS* and ST14. **(F)** Venn diagram of K2, *irp2*, *iroN* and *iucA*. **(G)** Venn diagram of K1, *irp2*, *iroN* and *iucA.* Such relationships were shown in 521 *K. pneumoniae* strains.

Among the 91 strains harboring *wzy-K1*, *p-rmpA*, *p-rmpA2* or *c-rmpA/A2*, 49 (53.8%) possessed *p-rmpA* and *p-rmpA2*, 18 (19.8%) possessing *wzy-K1*, *p-rmpA* and *p-rmpA2*, 15 (16.5%) possessing merely *p-rmpA2*. **Figure 3A** showed strong relationships among *wzy-K1*/*p-rmpA* or *p-rmpA*/*p-rmpA2*. In the 520 strains positive in *entB*, *irp2*, *iroN* or *iucA*, 278 (53.5%) harbored *entB* and *irp2*, 241 (46.3%) harboring only *entB*, 35 (6.7%) harboring all the four genes. **Figure 3B** showed strong relationships between *iucA*/*iroN* and *irp2*. Other relationships were also shown in: **Figure 3C** (K1, *peg-344*, *allS* and ST23), **Figure 3D** (K2, *p-rmpA*, *p-rmpA2* and *c-rmpA/A2*), **Figure 3E** (K2, *peg-344*, *allS* and ST14), **Figure 3F** (K2, *irp2*, *iroN* and *iucA*) and **Figure 3G** (K1, *irp2*, *iroN* and *iucA*). Gene *wzy-K1* was completely restricted to K1 serotype (31/31), vice versa. High prevalence of *peg-344* and *allS* was found in K1 strains (22/31, 28/31), but rarely in K2 ones (10/38, 0/38). Gene *allS* was mainly found in K1 strains (28/33), contrary to *peg-344* (22/65). K1 strains mostly belonged to ST23 (23/31) while less than a half (17/38) of K2 ones belonged to ST14. K1 strains showed higher rates of *rmpAs* (*p-rmpA*/*p-rmpA2*/*c-rmpA*/*A2*) and siderophore genes (*iroN*/*iucA*) than K2 ones: 23/31 vs 10/38 (*p <* 0.0001), 23/31 vs 9/38 (*p <* 0.0001), which “confirmed” hypervirulence in K1 strains.

According to the aforementioned criteria, 94 (18.0%), 165 (31.7%) and 29 (5.6%) strains were denoted as hypervirulent *K. pneumoniae* (HvKP), *bla*_KPC_(+)-KP and Hv-*bla*_KPC_(+)-KP, as shown in **Figure S1**. Consequently, 427 (82.0%) strains were cKP. Hv-*bla*_KPC_(+)-KP shared 17.6% (29/165) among *bla*_KPC_(+)-KP. For the *bla*_KPC_(+)-KP, ST11 accounted for 34.5% (57/165) while clonal group 258, including ST11, ST258, ST340 and ST437, was positive for 65.5% (108/165), indicating the focus of *bla*_KPC_(+)-KP.

### Distributions of Virulence Genes in Hv-*bla*_KPC_(+)-KP

**Figure 4:**
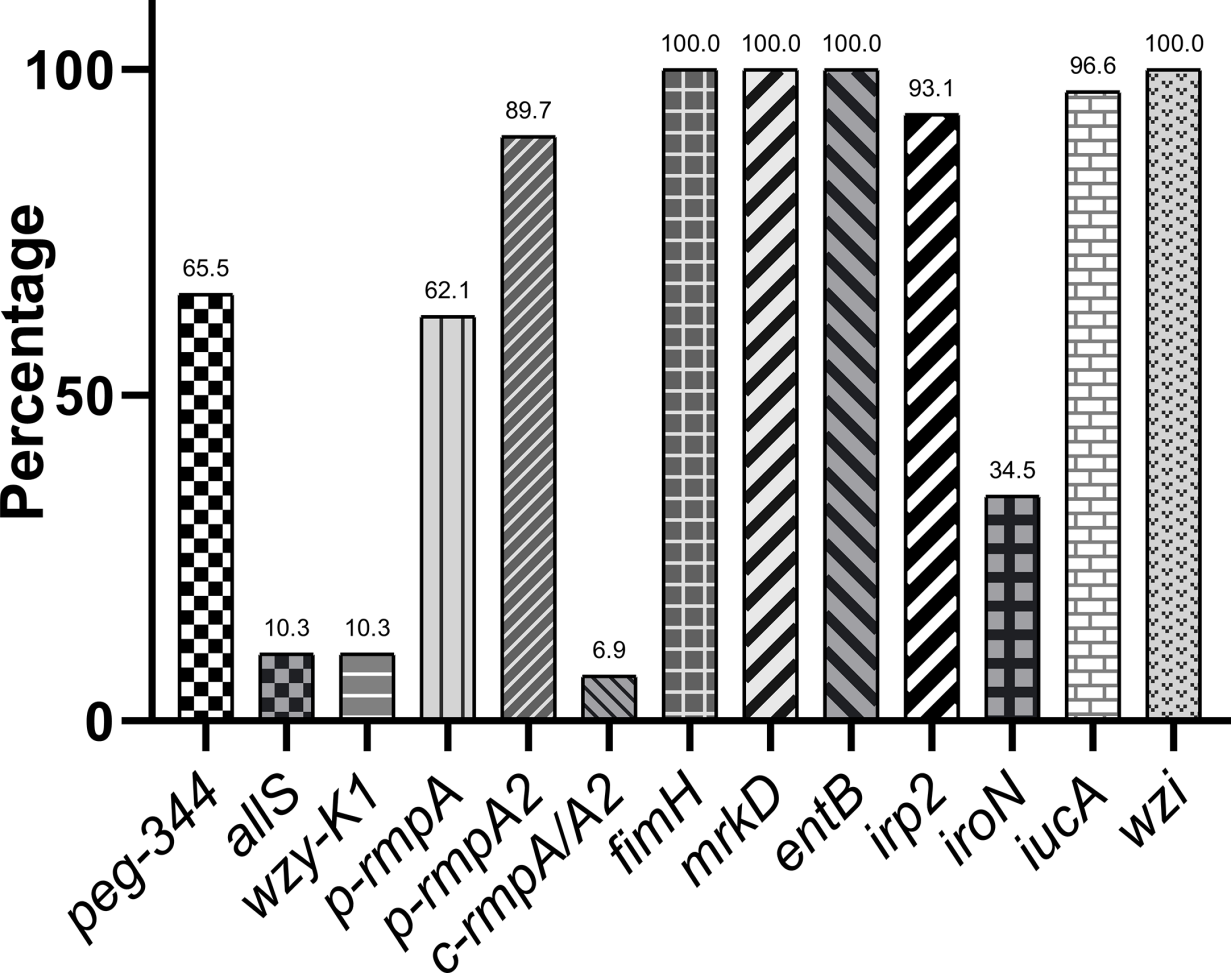
Distributions of virulence genes in Hv-*bla*_KPC_(+)-KP.

**Figure 4** presented greatly different prevalence of virulence genes in 29 Hv-*bla*_KPC_(+)-KP strains, ranging from *fimH* (100.0%), *mrkD* (100.0%), *entB* (100.0%), *wzi* (100.0%) to *c-rmpA/A2* (6.9%). Genes *iucA*, *p-rmpA2* and *p-rmpA* were positive in 28 (96.6%), 26 (89.7%) and 18 (62.1%) Hv-*bla*_KPC_(+)-KP strains respectively. A sum of 28 (96.6%) strains presented ≥ 3 siderophores and 29 (100.0%) carried *p-rmpA/p-rmpA2* (*p >* 0.9999).

**Figure 5:**
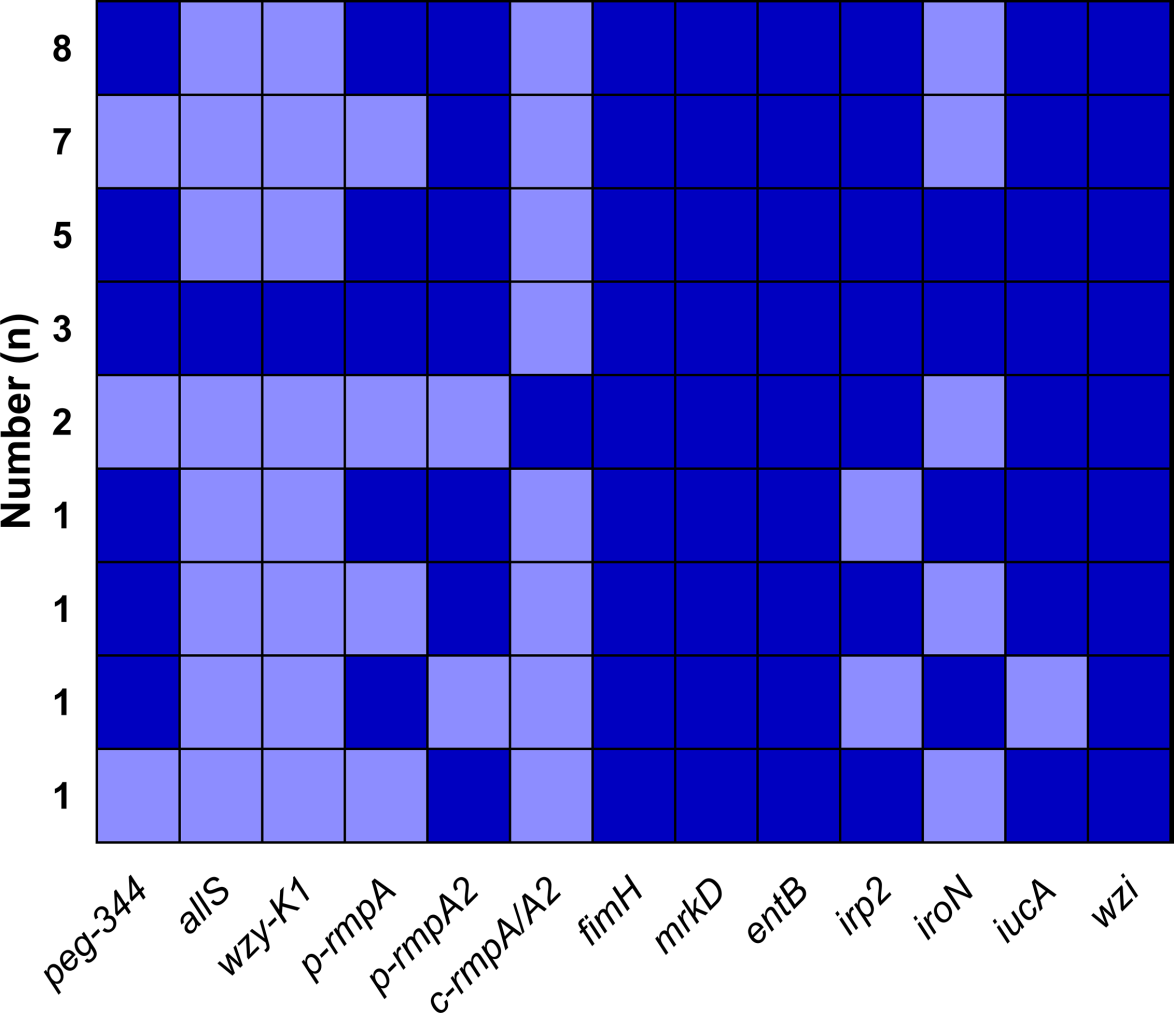
Modes of virulence genes in Hv-*bla*_KPC_(+)-KP. The presence of virulence genes is represented by a dark blue box and the absence of others is represented by a light blue box.

A total of nine modes of virulence genes were found among the 29 Hv-*bla*_KPC_(+)-KP strains, as shown in **Figure 5**. And the first four modes consisted of eight (27.6%), seven (24.1%), five (17.2%) and three (10.3%) strains, which constituted the majority.

### Distributions of STs and Serotypes in Hv-*bla*_KPC_(+)-KP

**Figure 6:**
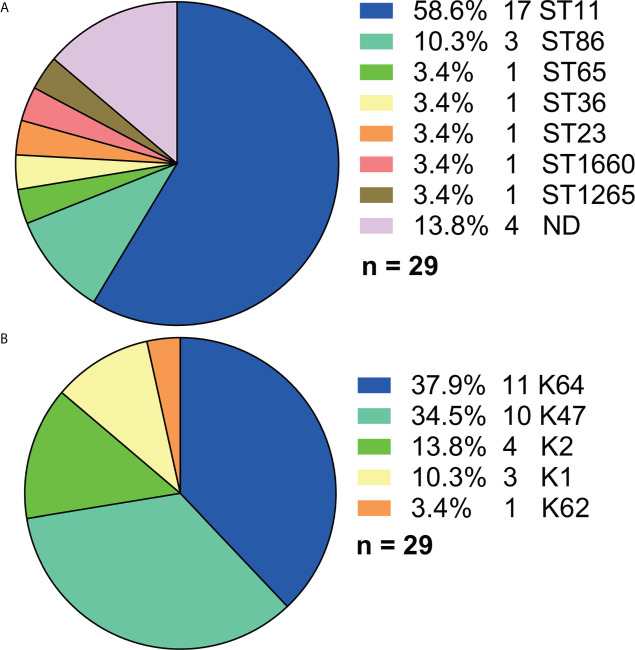
Distributions of STs and serotypes in Hv-*bla*_KPC_(+)-KP. **(A)** Distribution of STs in Hv-*bla*_KPC_(+)-KP. **(B)** Distribution of serotypes in Hv-*bla*_KPC_(+)-KP. Statistics were made among the 29 Hv-*bla*_KPC_(+)-KP strains. ND, not defined.

Among the 29 Hv-*bla*_KPC_(+)-KP strains, ST11 accounted for the majority (17, 58.6%) although more than 10 STs were found in total (**Figure 6A**). And five serotypes were found (**Figure 6B**), among which K64 (11, 37.9%) and K47 (10, 34.5%) made the majority.

### Locations of Virulence and *bla*_KPC_ Genes in Hv-*bla*_KPC_(+)-KP

**Figure 7:**
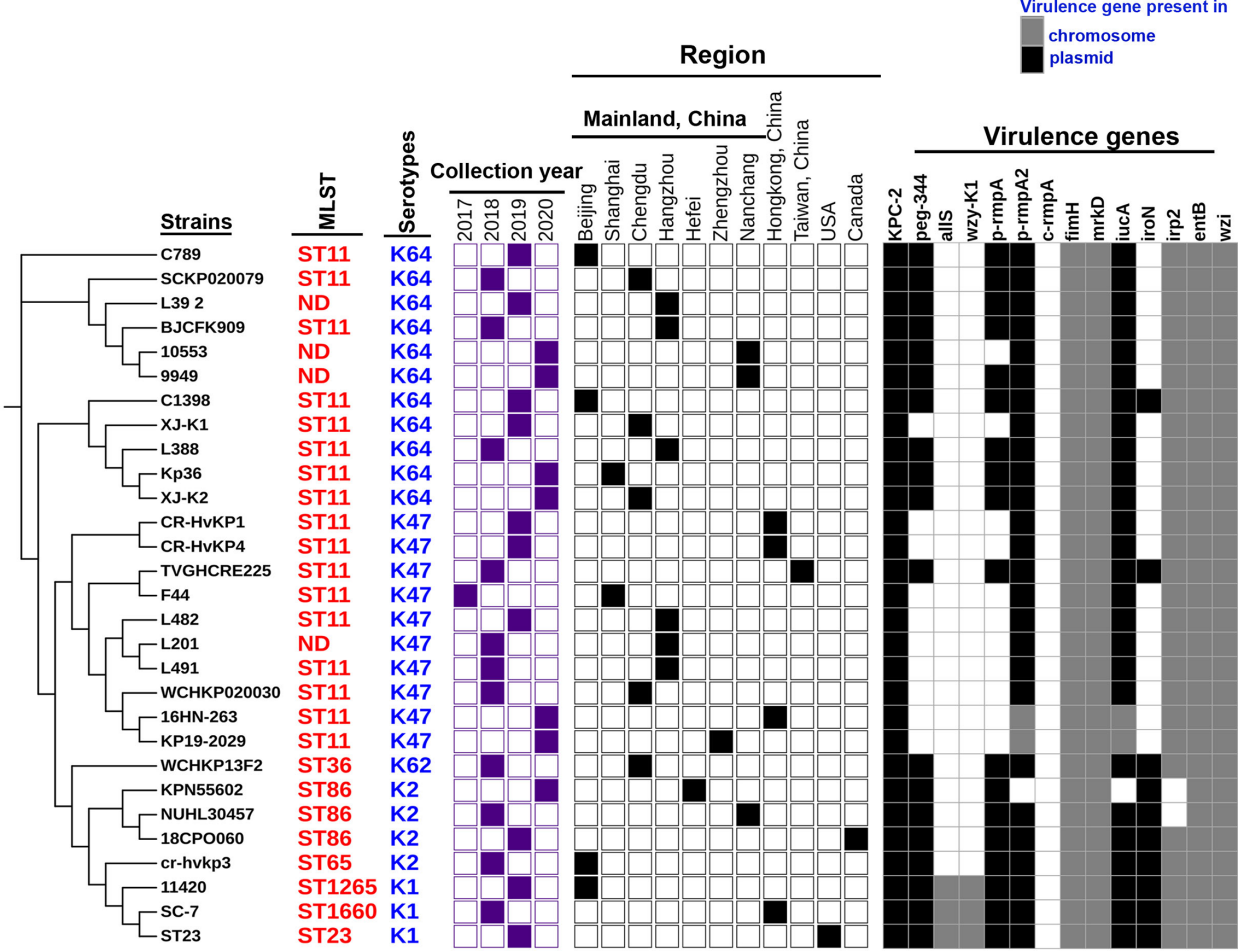
Characteristics of the 29 Hv-*bla*_KPC_(+)-KP strains. The phylogenetic relationship of the 29 Hv-*bla*_KPC_(+)-KP strains was analyzed by kSNP 3.0 Based on the predicted results, the binary gene presence/absence matrix was created reflecting the collection year, collection region, *bla*_KPC-2_ gene and core virulence genes. The STs of Hv-*bla*_KPC_(+)-KP strains were marked on the right of the phylogenetic tree. ND: not defined. The presence of genes, etc. is represented by a solid box. and the absence of others is represented by a white box.

**Figure 8:**
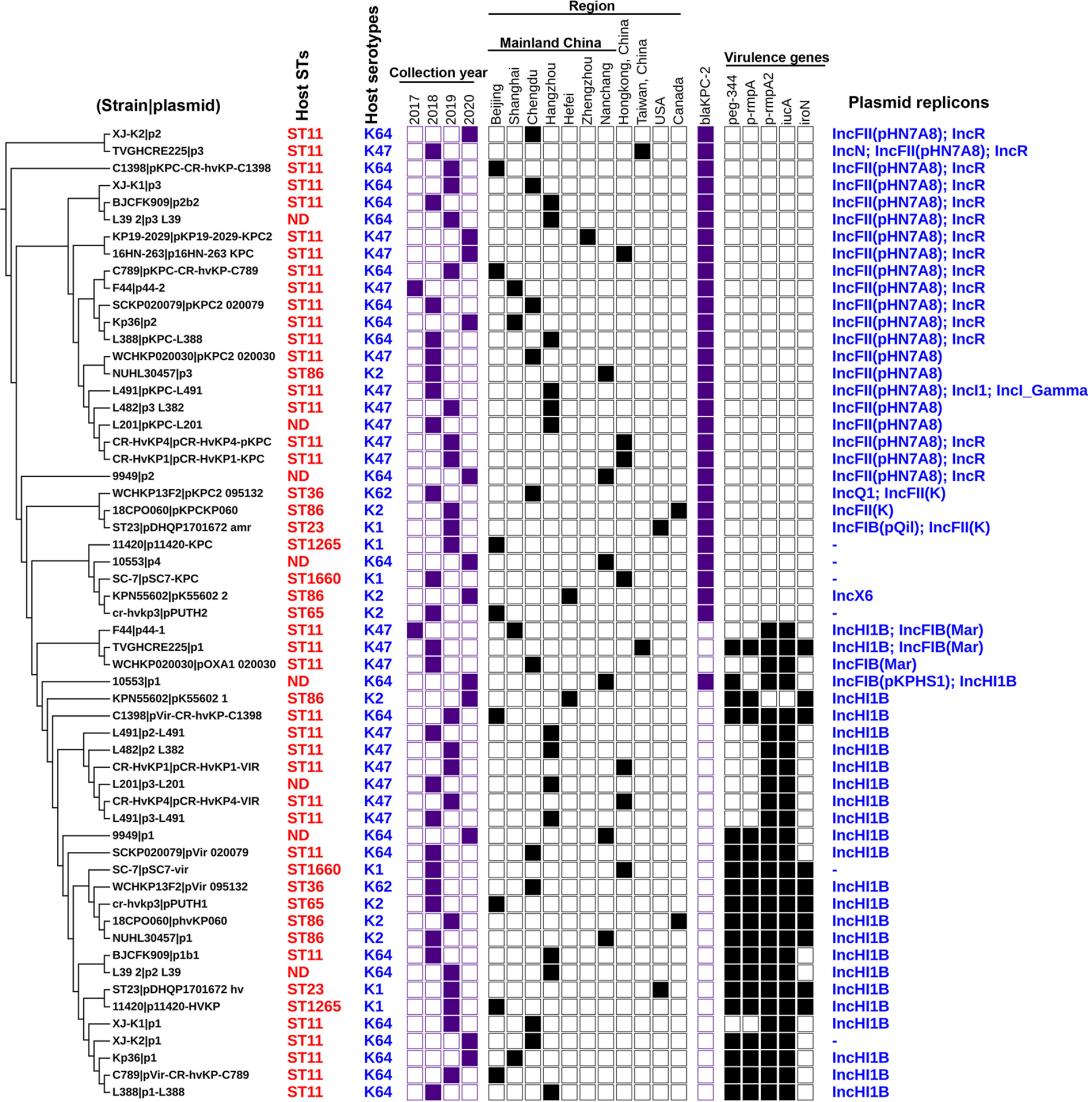
Details for the characteristics of the 57 antibiotic-resistance or virulence plasmids carried by the 29 Hv-*bla*_KPC_(+)-KP strains. The phylogenetic patterns were based on the presence/absence of orthologous gene families of 57 plasmids under analysis. Seven categories of information were presented in this figure, including the phylogenetic tree of 57 plasmids, STs of host strains, collection year, collection region, *bla*_KPC-2_ gene, core virulence genes and replicon types of plasmids. ND, not defined. The presence of genes, etc. is represented by a solid box and the absence of others is represented by a white box.

Trends in virulence among Hv-*bla*_KPC_(+)-KP infections revealed that the prevalence of Hv-*bla*_KPC_(+)-KP significantly increased between 2018 and 2020, mainly from China, especially Mainland China (**Figure 7**). We found that IncHI1B plasmids were predominantly responsible for the virulence genes (23 strains, 79.3%) and IncFII plasmids were the main contributors for the gene *bla*_KPC_ (23 strains, 79.3%), suggesting that Hv-*bla*_KPC_(+)-KP strains were mainly induced by two different plasmids (**Figure 8**). IncHI1B and IncFII plasmids constituted the alarmingly successful combination among Hv-*bla*_KPC_(+)-KP strains. ST11 accounted for 17 (58.6%) among the 29 Hv-*bla*_KPC_(+)-KP strains. Those Hv-*bla*_KPC_(+)-KP strains with ST11 typically corresponded to K47 (9/17) and K64 (8/17) serotypes and were divided into four super subgroups. Those with ST86 were all K2 serotype (4/4).

## Discussion

This study investigated the general distributions of key virulence genes in *K. pneumoniae*, in particular Hv-*bla*_KPC_(+)-KP.

Among the 521 strains, 65 were positive for *peg-344*, of which 63 were denoted as HvKP. A sensitivity of 96.9% was therefore yielded, similar as the report (*p* = 0.5791) (Russo et al., 2018). Gene *allS* was not restricted to K1 and K2 strains, different from the document (Yu et al., 2008). The reason may lie in the different specimen types of analyzed strains. Gene *wzy-K1* (formerly designated *magA*), corresponding to K1 serotype, vice versa, could help *K. pneumoniae* yield macromolecular EPS, which confers hypervirulence (Fang et al., 2004). Wzi is a protein riveting capsular polysaccharides, loss of which *K. pneumoniae* should be acapsular (Rahn et al., 2003). Acapsule was found in 13 (2.5%) strains, which means low virulence. A total of four kinds of siderophores were found in *K. pneumoniae* strains: enterobactin, salmochelin, yersiniabactin, and aerobactin (Russo and Marr, 2019). Intriguingly, one strain (strain AR_0096, accession number: CP027612.1) was found for none siderophore, indicating other ferric uptake systems than siderophores may also provide a certain amount of iron for growth and reproductivity (Hsieh et al., 2008).

Except for macromolecular EPS and excessive siderophores, hypercapsule could also contribute to hypervirulence (Russo and Marr, 2019), which is typically conferred by *p-rmpA*, *p-rmpA2* or *c-rmpA/A2* genes. Hypercapsule played an equal role with excessive siderophores (15.4 vs 16.3%, *p* = 0.6714) in hypervirulence of *K. pneumoniae*. The reason lies in the same pLVPK-like plasmids harboring *rmpA*s and siderophore genes concurrently.

Gene *bla*_KPC_ was first reported from USA in 1996 (Yigit et al., 2001). Then, the first *bla*_KPC-2_(+)-KP strain was reported in mainland China in 2007 (Wei et al., 2007). CRKP has now shared 70 – 90% of carbapenem-resistant *Enterobacteriaceae* in the European Union and China (Grundmann et al., 2017; Zhang et al., 2017b). To date, *bla*_KPC_ consists of more than 50 subtypes, among which *bla*_KPC-2_ is the most successful one and predominates CRKP worldwide. *bla*_KPC-2_ was positive in 132 (25.3%) strains while *bla*_KPC-3_ was found in 30 (5.8%) strains. Our study also showed clonal group 258 but not ST11 made up the majority of *bla*_KPC_(+)-KP (Wang et al., 2018; Fu et al., 2019); The reason comes from the global distribution of the 521 strains.

The first Hv-CRKP, belonging to K2 and ST65, was unveiled in mainland China in 2015, which was isolated from blood in Wuhan City in March 2013 (Zhang et al., 2015). Armed with hypervirulence and extreme drug-resistance, Hv-CRKP causes greater mortality and becomes notorious (Gu et al., 2018). Our study showed a positive rate of 5.6% for Hv-*bla*_KPC_(+)-KP worldwide. Different prevalence of *iucA*, *p-rmpA2* and *p-rmpA* in Hv-*bla*_KPC_(+)-KP strains suggested their different roles in hypervirulence. The modes of virulence genes were rather diverse in Hv-*bla*_KPC_(+)-KP. Similar prevalence of ≥ 3 siderophores and *p-rmpA/p-rmpA2* (*p >* 0.9999) indicated their equal roles in hypervirulence of Hv-*bla*_KPC_(+)-KP strains, which also originated from the same pLVPK-like plasmids harboring *rmpA*s and siderophore genes simultaneously. The proportion of K64 was (11, 37.9%), lower than another report (Zhang et al., 2020) (*p <* 0.0001). Further, IncHI1B plasmids carrying virulence genes and IncFII ones with *bla*_KPC_ were responsible for both 23 strains, suggesting IncHI1B and IncFII plasmids jointly constitute the most successful combination. Furthermore, the phylogenetic trees revealed that the 29 Hv-*bla*_KPC_(+)-KP strains belonged to four super clusters although three clusters all possessed ST11 strains.

Hv-*bla*_KPC_(+)-KP evolution may occur through two mechanisms. The first pathway is *via* HvKP acquiring a plasmid carrying drug-resistance determinants (Wei et al., 2016; Feng et al., 2018) or by the insertion of resistance genes into virulence plasmid or chromosome harbored by HvKP (Zhang et al., 2016; Fu et al., 2018). The second pathway is *via* multidrug-resistant/extreme drug-resistant cKP acquiring a pK2044- or pLVPK-like virulence plasmid or integrated virulence genes into drug-resistance plasmids (Gu et al., 2018). Our data showed it was most likely that Hv-*bla*_KPC_(+)-KP mainly evolved through the second pathway, i.e. *via bla*_KPC_(+)-KP acquiring another plasmid harboring virulence genes. Zhou et al. (2020) and Tang et al. (2020) preached that CRISPR-Cas system deficiency in ST11 may play a vital role. However, the two papers elucidated only *bla*_KPC_ entering ST11 strains; IncHI1B plasmids are different from IncFII ones: rare protospacers were found and they lacked Type IV secretion systems, e.g. *traM* gene. Therefore, the mechanisms behind IncHI1B plasmids entering ST11 strains would be sophisticated and intriguing.

This study has some limitations. First, the specimen types of 521 *K. pneumoniae* strains are not well known. Second, some positive virulence genes do not inevitably mean “exact” hypervirulence.

Taken together, positive rates of virulence genes vary overwhelmingly in *K. pneumoniae*. Hypercapsule plays an equal proportion with excessive siderophores in hypervirulence of *K. pneumoniae*. Virulence genes *iucA*, *p-rmpA2* and *p-rmpA* are primary ones inducing Hv-*bla*_KPC_(+)-KP. IncHI1B plasmids carrying virulence genes and IncFII ones with *bla*_KPC_ constitute the primary combination responsible for Hv-*bla*_KPC_(+)-KP. Hv-*bla*_KPC_(+)-KP urges more insightful investigations.

## Data Availability Statement

Publicly available datasets were analyzed in this study. This data can be found here: https://pan.baidu.com/s/1sbsl_phsx8IRoQeeY87e-w (password: xf5l).

## Author Contributions

DH, YL and PR conceived the study. DT, WC, PF, WW and XJ collected the 521 genomes. DH, YL, PR and XL did bioinformation analysis. DH and YL wrote the manuscript, which was revised by XL and XJ. All authors contributed to the article and approved the submitted version.

## Funding

This work was funded by research grants from the National Natural Science Foundation of China (grants 81871692, 81572031, and 82002170) and the Shanghai Municipal Science and Technology Commission (grant number 19JC1413002).

## Conflict of Interest

The authors declare that the research was conducted in the absence of any commercial or financial relationships that could be construed as a potential conflict of interest.
