# Supplementary material for: Molecular Epidemiology of Hypervirulent Carbapenemase-Producing Klebsiella pneumoniae
Source: Front Cell Infect Microbiol. 2021 Apr 7;11:661218. doi: 10.3389/fcimb.2021.661218 (PMC8058458; doi:10.3389/fcimb.2021.661218)
Supplement: Supplementary file 1 [file DataSheet_1.docx]

**Molecular epidemiology of hypervirulent carbapenemase-producing *Klebsiella pneumoniae***

**SUPPLEMENTARY DATA**

**Table S1.** Information of the 521 complete whole genomes (Table S1) of K. pneumoniae from the GenBank. (the Excel file named Table S1.xlsx)

**Table S2.** The virulence genes of *K. pneumoniae* with experimental supports used in this study.

**Figure S1.** Distributions of HvKP, *bla*_KPC_(+)-KP and Hv-*bla*_KPC_(+)-KP in 521 *K. pneumoniae* strains.

**Table S2.** The virulence genes of *K. pneumoniae* with experimental supports used in this study.

| **Gene name** | **Accession number** | **Host strain** | **Location** |
| --- | --- | --- | --- |
| *wzy-K1* | AP006725.1 | NTUH-K2044 | chromosome |
| *allS* | AP006725.1 | NTUH-K2044 | chromosome |
| *entB* | AP006725.1 | NTUH-K2044 | chromosome |
| *irp2* | AP006725.1 | NTUH-K2044 | chromosome |
| *iroN* | AP006726.1 | NTUH-K2044 | plasmid |
| *iucA* | AP006726.1 | NTUH-K2044 | plasmid |
| *fimH* | AP006725.1 | NTUH-K2044 | chromosome |
| *mrkD* | AP006725.1 | NTUH-K2044 | chromosome |
| *c-rmpA* | AP006725.1 | NTUH-K2044 | chromosome |
| *p-rmpA2* | AP006726.1 | NTUH-K2044 | plasmid |
| *p-rmpA* | AP006726.1 | NTUH-K2044 | plasmid |
| *peg-344* | AP006726.1 | NTUH-K2044 | plasmid |
| *wzi* | AP006725.1 | NTUH-K2044 | chromosome |


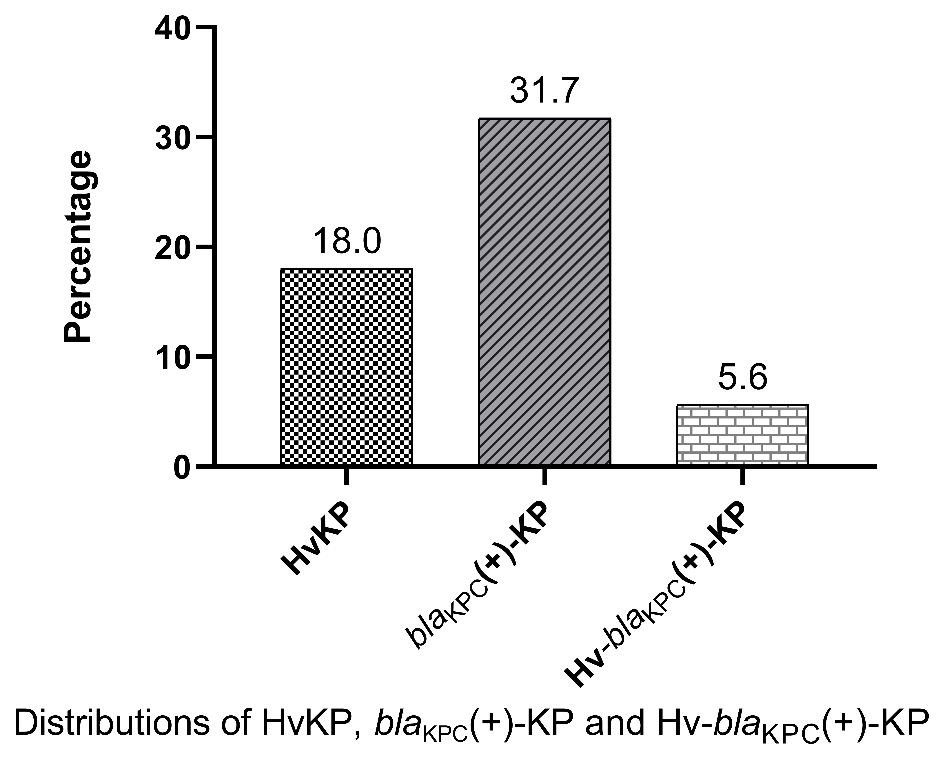


**Figure S1**
